# The Renewable Energy Landscape: Power‐to‐X, Land Competition, and Ecological Balance in Denmark

**DOI:** 10.1002/gch2.70132

**Published:** 2026-07-16

**Authors:** Clara Donahue, Aggelos Sotiropoulos, George Xydis

**Affiliations:** ^1^ Department of Business Development and Technology Aarhus University Herning Denmark; ^2^ School of Chemical Engineering National Technical University of Athens Zografou Greece; ^3^ Department of Mechanical Engineering University of the Peloponnese Patras Greece

**Keywords:** balance of nature, community engagement, economic impact analysis, energy security, environmental impact assessment, environmental planning, renewable energy, social equality, social impact assessment, stakeholder analysis

## Abstract

As Denmark continues to move towards a more decarbonized future, Power‐to‐X (PtX) technologies are becoming an important part of green energy and fuel. These technologies also have significant impacts on land and social equity. This study combines spatial analysis, qualitative case studies, and stakeholder mapping to analyze how PtX expansion interacts with land allocation and existing socio‐economic inequalities within Denmark. Spatial mapping using geographical information systems (GIS) reveals that PtX infrastructure is more heavily sited in rural areas, heightening competition for land, environmental impacts, and economic impacts on local communities. These burdens affect rural communities more than urban ones. Case studies of three PtX facilities highlight how different planning and community engagement strategies produce varying outcomes. Stakeholder analysis identifies key tensions between developer actions and public response. Findings show that, while PtX can improve Denmark's energy security, a proactive policy is needed to ensure benefits are shared equally and community concerns are addressed. Recommendations include mandating early community engagement, prioritizing existing industrial sites, strengthening local benefit‐sharing programs, enhancing environmental and social impact assessments, and expanding community education. This approach is crucial for Denmark to integrate PtX at scale while addressing spatial and socio‐economic tradeoffs.

## Graphical abstract

The Anatomy of Land Use.

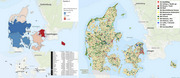

## 1 Introduction

As Denmark continues to be a leader in the development of a more sustainable and decarbonized energy system, innovative technologies such as Power‐to‐X (PtX) are being explored. PtX is a relatively new technological development, focusing on the use of excess energy produced from renewable energy technologies to power other processes, such as hydrogen production, green fuel creation, etc. This technology may play a crucial role in achieving net‐zero emissions through a more resourceful and innovative energy grid.

The land use demands required to implement these PtX technologies pose new development opportunities and challenges. Spatial demands for technology such as wind and solar farms may create land use conflicts for local communities. Changes to the existing infrastructure and environments have the potential to create new land use and energy inequalities, while worsening existing ones, with environmental and social changes having the greatest impact. While PtX will be an important part of Denmark's future energy grid, the need to address these issues will continue to intensify.

This study will aim to confront and analyze land allocation and social equity implications associated with the development of PtX technologies. This research will focus on hydrogen production and grid electrification. An analysis of current land use patterns throughout Denmark, public and professional perspectives, and existing case studies will be explored. The purpose of this research is to develop an actionable plan/strategy that will help Denmark meet its PtX goals while also addressing the associated land and social conflicts. The research will employ a mixed‐methods approach, combining geospatial analysis with qualitative insights from stakeholder engagement. The ultimate objective is to foster a just and equitable transition, ensuring that the path to a sustainable future does not come at the expense of community well‐being or environmental integrity.

Power to X is a rising technology that uses surplus renewable electricity to power other processes or produce different forms of energy. “X” is a representation of the many different power outputs that can be produced using surplus renewable electricity, such as heat, hydrogen, fuels, etc. There are different PtX uses that have the most promise of expansion and use in a more sustainable future. Power‐to‐Hydrogen is the usage of excess renewable electricity generated from forms such as wind and solar to create hydrogen through electrolysis. Surplus energy from the original energy process is used to power electrolysis plants, converting water to pure hydrogen. Power‐to‐Fuel is an offset of the power to hydrogen process, where hydrogen produced through electrolysis can be combined with carbon dioxide to create synthetic fuels such as methane. Power‐to‐Liquid has similar applications to power‐to‐fuel; when CO_2_ and hydrogen are combined, they can also produce liquid fuels that have the potential to power planes, ships, etc. Power‐to‐Chemicals is the use of hydrogen and CO_2_ produced through the previous processes to create industrial chemicals such as ammonia. Lastly, Power‐to‐Heat is the use of excess electricity generated from renewable sources to power electricity grids [1, 2].

Communities may have issues with the implementation of Power‐to‐X because of fears around environmental impacts and landscape changes. High costs for implementation may raise concerns about prioritization of industrial endeavors versus the needs of local communities and businesses. High costs may also lead to public resistance due to funding concerns. The large amounts of energy inputs required to power these processes may divert energy usage from more critical communities, raising energy prices and possibly widening existing energy inequalities. PtX installations may create competition for other sectors such as agriculture and urban development. There may be concerns about the disruption of local ecosystems and wildlife habitats [3]. These concerns may be mitigated using proper site selection and environmental impact assessments. However, public concerns may create resistance. PtX processes, such as power to hydrogen, that require large amounts of water, may also create issues for available water usage [4]. Addressing these issues is important to the development of these projects and the surrounding communities affected by them.

## 2 Literature Review

A review of existing research was conducted to understand the current state of Denmark's land use and energy grid transitions. The review focused on the demands of PtX technologies, such as wind, solar, and hydrogen production, and how these technologies compete for land availability and their impact on other sectors. This paper also looked at previous studies regarding the socio‐economic impacts of energy transitions and how they may have affected access to energy or how such changes have affected existing energy inequalities.

### 2.1 Land Use and Infrastructure

There is a growing amount of literature about land demands and conflicts associated with the development of renewable energy projects. PtX projects, particularly in relation to the production of hydrogen and grid electrification, require large amounts of available land for the development of these projects. The land required for PtX installations varies depending on the project and the type of renewable energy source being used. The Danish Energy Agency has stated that the integration of these PtX technologies requires the consideration of both technical feasibility and compatibility of land [5]. Lovering et al. explored the average land usage of these projects, finding that onshore wind and solar farms often require the largest amount of land use for project implementation [6]. In Denmark, competition for the use of the land is often more severe in rural areas. Wind farms, solar parks, even plans for PtX and direct air capture (DAC) projects should fit. PtX projects are more favorable in rural areas, due to their ideal wind conditions, such as proximity to water, and available space. Recent studies, such as Scheyl, found that spatial inequalities are more prominent in rural communities due to the development of energy infrastructure [6]. Urban communities, however, were found to benefit more from these developments with growing economic benefits, while rural communities face harsher social and environmental‐related costs. Rural areas also face greater challenges with ecosystem disruption from large‐scale projects, as found in Eveloy et al. [2]. These effects create more socio‐economic disparities within rural communities because of these large energy projects compared to more urban locations.

### 2.2 Social Equity

The transition to these technologies raises the issue of energy justice and its effects on social equity. According to Jenkins et al., energy justice refers to the fair distribution of all benefits and burdens associated with energy production and consumption [7]. A disproportionate distribution of either leads to energy injustice throughout communities. Zhou et al. identified issues within rural areas of Denmark that already face social and economic challenges and found that they are more likely to be negatively impacted by land use disruption and degradation resulting from energy infrastructure development [8]. These issues support the notion that more rural areas of the country are at a higher risk of facing project‐related impacts within their communities. Zhou et al. also explored the effects these projects have on more urban areas in Denmark, citing that urban communities are more likely to reap the economic benefits and energy access compared to rural regions. Sovacool et al. also suggest that urban areas are more likely to benefit from energy investments through job creation and more efficient infrastructure, while rural areas end up facing higher costs and more general downsides to these projects [9]. Heffron et al. added to the idea that marginalized groups should be more included in the decisions related to energy projects to combat energy injustices. In the scope of Denmark's energy infrastructure, this would mean more community involvement from rural areas in decisions regarding PtX technology and their infrastructure [10, 11].

### 2.3 Policy Mitigation

Addressing issues with the allocation of land and energy injustices related to the implementation of these projects requires changes in policy [12, 13]. Including areas that are disproportionately affected by these energy decisions should be essential to the decisions and policies created in the future [14]. Community agreements and proper financial compensation in these areas may also help to offset energy inequalities and injustices related to these issues. Zoning regulations are also identified in current literature to identify areas for renewable energy infrastructure and to help minimize conflicts between other sectors, such as agriculture and housing. Thorough Environmental Impact Assessments (EIA) have also been identified as an important strategy to avoid environmental degradation [14]. Existing literature shows that there are many different avenues that can be explored in the discussion of policy changes, with the main takeaway being the need to include all communities in the decision‐making process as well as the distribution of proper compensation.

## 3 Methodology

This study uses a mixed methods approach to the research methodology. Both spatial and literary data were used to assess the possible social‐political inequalities and land use impacts associated with the integration of PtX technologies in Denmark [15]. This research approach offers a comprehensive understanding of how these PtX technologies may alter Denmark's existing land use patterns and energy inequalities. The combination of these methodologies allows this study to provide a broader understanding of the challenges that Denmark will face in their energy transition, considering different criteria for PtX project selection. These criteria provide a structured framework for evaluating potential project locations and designs, ensuring they align with both national energy goals and local community needs. Selection criteria can be environmental, such as prioritizing sites with low biodiversity value or proximity to existing renewable energy infrastructure to minimize new land take. They can also be social, for example, requiring projects to demonstrate community benefits or avoid areas with high landscape and cultural significance. Furthermore, technical and economic criteria, like access to water sources for hydrogen production or connection to the electricity and gas grid, are fundamental to any project's viability. A focus on actionable recommendations to policymakers and communities will be the end goal of combining all methods within this study.

To ensure the rigor of the qualitative components (document reviews and stakeholder analysis), a systematic approach was applied. A total of 33 documents were reviewed across the three case studies, comprising 12 official project reports or EIAs, 14 local and national media articles, and 7 municipal planning documents. Coding procedures involved an initial open coding phase to identify emerging themes (e.g., “land conflict,” “community benefit”), followed by axial coding to group these into the core categories presented in the stakeholder tables (Interests, Concerns). To validate the findings, a triangulation protocol was used, comparing insights across the three different data sources (media, official documents, spatial data) to confirm patterns and ensure that no single source influenced the conclusions.

## 4 Spatial Analysis

Geographical Information Systems (GIS) were used to map existing land use patterns in combination with proposed PtX infrastructure. This geospatial data was used to identify areas of overlap in existing land use with proposed future PtX projects, such as wind, solar, and hydrogen production. Existing GIS maps from previous studies were used to display relevant information regarding existing infrastructure and land use [16]. ArcGIS was used to map locations of upcoming PtX project installations throughout Denmark. Potential areas of land use conflicts were also identified through observations of where energy infrastructure will overlap with existing land use sectors.

### 4.1 Secondary Layers

A tile layer depicting land use in Denmark was set as a base layer for the map [17]. The layer contained 13 different land use options ranging from urban, road, forest, sea, etc. This layer was essential to getting a broad view of Denmark's current use of land and the differences between areas of high urban development and more rural use.

### 4.2 Primary Layers

A point layer was created using Innargi Projects, Green Together, and the Danish Energy Agency to identify PtX projects that are currently being built or scheduled to be built within Denmark. Each point represents a new or soon‐to‐be‐existing PtX site, including hydrogen production, solar and wind for grid electrification, geothermal for grid electrification, etc. A master list of each project, its description, and coordinates was made in Excel. The Excel document was then saved as a CSV file and uploaded to ArcGIS as a point layer. This layer is important in identifying new and future infrastructure, providing an overview of Denmark's regional and nationwide PtX plans. iii. GIS Analysis

A feature layer was added to depict the population of Denmark. The field types were set to show the ratio of total population to population density. The ratio was color‐coded from blue to red, with blue being a larger ratio of population to density and red being a smaller ratio [18].

## 5 Case Studies

A range of case studies were analyzed to understand the effects and implications that PtX projects/infrastructure have on land use and socio‐economic inequalities. An analysis was conducted of the ongoing and completed PtX projects in Denmark with a focus on hydrogen production through electrolysis and grid electrification.

A review was conducted on similar energy transitions within other regions/countries. This analysis provided information on the obstacles faced and the mitigation strategies of such a transition. The following projects were used as case study subjects:

- Kassø PtX Facility: Southern Denmark
- Lille Torup: Jutland, Denmark
- Fjord PtX: Western Denmark

For each case study, data were collected through the following methods:

‐ *Review of Primary Documents and Media*: Official project reports, impact assessments, and articles released by the press and local media were used to gain an understanding of each project and the general public opinion surrounding it. To capture public and stakeholder perspectives, a review of local and national media articles was conducted, focusing on opinion pieces, letters to the editor, and news reports covering community meetings or protests. This press analysis was supplemented by reviewing publicly available social media discussions and statements from local community groups or NGOs. These were cross‐referenced with municipal planning documents and permit applications to understand the formal regulatory context.
‐ *Review of Secondary Documents*: Existing academic studies analyzing these types of projects were used to identify patterns of major issues and insights.
‐ *Use of Spatial Data*: GIS data showing project sites, existing infrastructure, and current land use patterns were overlaid with case‐specific data to visualize conflicts and benefits.

## 6 Stakeholder Analysis

This stakeholder analysis draws exclusively from document review and media analysis, as primary interview data were beyond this study's scope. However, the triangulation of 33 documents across three data types (official reports, media coverage, municipal planning documents) enabled the identification of recurring stakeholder perspectives. For example, farmer opposition emerged in 12 distinct media sources across the Fjord and Lille Torup cases, and resident concerns about visual impacts appeared in 8 Kassø project documents. A qualitative review was conducted on the existing case studies and available public insights investigating PtX projects in local Danish communities [19]. Using this information, two tables were created to summarize the interests and areas of risk for these projects, including potential stakeholder groups and their areas of influence. A list of summarized case insights was also created to mitigate the issues highlighted in the stakeholder analysis. Stakeholder analysis was built on information identified within the case studies and was structured to map the key stakeholder groups, their interests, influence, and mitigation strategies. Stakeholder influence was assessed by applying a consistent framework to the information gathered from case study documents and media analysis. For each identified stakeholder group, influence was evaluated based on three key dimensions: their formal authority (i.e., power to approve permits), managing the essential resources (i.e., funding, grid access), and their social dimensions (i.e., ability to shape public opinion). By systematically analyzing the case studies for evidence of these dimensions – social opposition by local communities – the analysis could determine the stage of development where that influence is most impactful. The following factors were reviewed:

‐ *Stakeholder Groups*: Stakeholders were identified based on the direct and indirect influences they may have on PtX projects.
‐ *Identification of Interests*: Areas of interest for stakeholders were identified using structured thematic coding of the reviewed documents. This process involved systematically tagging text segments related to stakeholder concerns (such as visual impact, employment, and property values) and benefits, which are presented in Tables 1, 2, and 3.
‐ *Assessment of Influence*: Stakeholder influence was identified based on where each group may hold the most power in the process of PtX project development.
‐ *Cross‐Case Insights*: Comparison of stakeholder interactions within each case study helped to identify patterns, areas of interest/risk, and possible strategies of mitigation.

**TABLE 1:** Stakeholder breakdown of role, interest, and influence.

Stakeholder Group	Role in PtX Projects	Areas of Interest	Influence
Regional Government	In charge of zoning, approval of local plans, and local grid integration.	Areas may include jobs, land use, property taxes, and value, public opinion/interest.	Having a strong influence within the local jurisdiction can ultimately fight the development of a project.
National government	In charge of overall project strategy, permits, and EU regulations.	Areas may include climate targets, industrial and economic growth.	Can potentially slow or accelerate projects through permits, regulations, and funding.
Infrastructure operators	They are the technical backbones of grid connections, storage, and pipelines.	Areas may include the utilization of assets, public and worker safety.	Have a technical influence.
Local residents	They are the community and residents of the project site.	Areas of interest may include jobs, property tax and value, pollution (Visual, noise, environmental), water use, and public safety.	Influence local government and social impact/perception.
Developers	They are the capital and choice of technology.	Timely development, social perception, and return on investment.	Have influence over project design.

**TABLE 2:** Areas of concern in PtX development.

Area	Approach by Case Studies	Areas of Risk/Concern
Economic Development	‐ Kassø Facility's surplus heat ‐ Fjord project local job creation	Inequal distribution of economic benefits between rural and urban groups.
Environmental Impact and Land Use	‐ Lille Torup's use of existing pipelines	Land competition and loss of habitat/environmental disruption.
Social Acceptance	‐ Kassø project's community engagement early into development	Safety, environmental changes, and property disruption.

**TABLE 3:** Mitigation Strategies.

Action	Reasoning and Case Evidence
1 Early Action Participation	Have an open dialogue with residents prior to site selection. ‐ Positive outcome in Kassø ‐ Negative results seen in Fjord PtX from limited early engagement.
2 Incorporate Community Benefit Packages	Consider how project development can be used to positively influence the lives of host residents. ‐ Use of surplus heat in Kassø ‐ Lille Torup PtX pledges to create new jobs
3 Prioritization of Existing Infrastructure	Prioritize the use of existing industrial brownfields and infrastructure. ‐ Lille Torup's use of existing pipeline corridors
4 Knowledge Hubs for Residents	Input programs that allow for transparency between developers and residents. Allow open monitoring of emissions, water, and land use, etc.

## 7 Results

**FIGURE 1:**
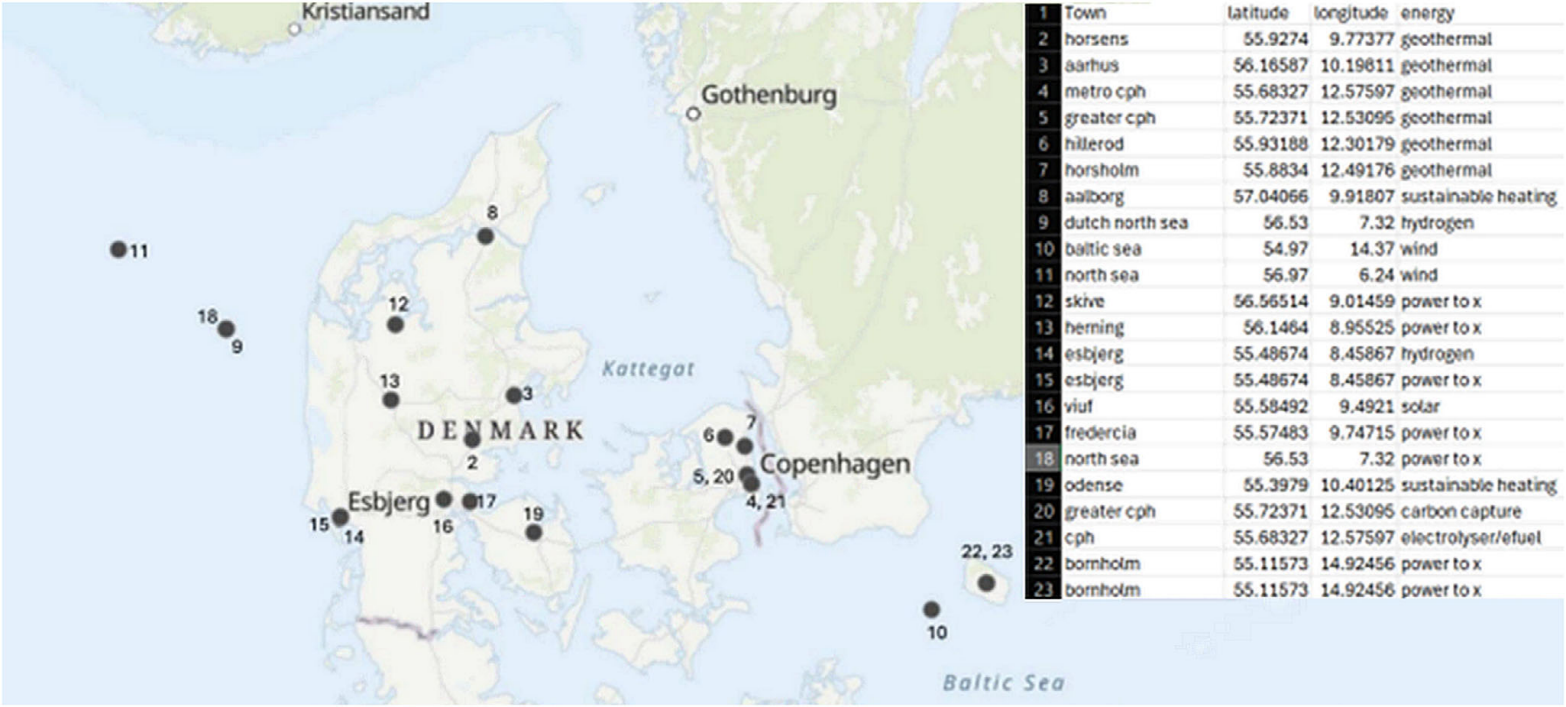
Future and new infrastructure.

**FIGURE 2:**
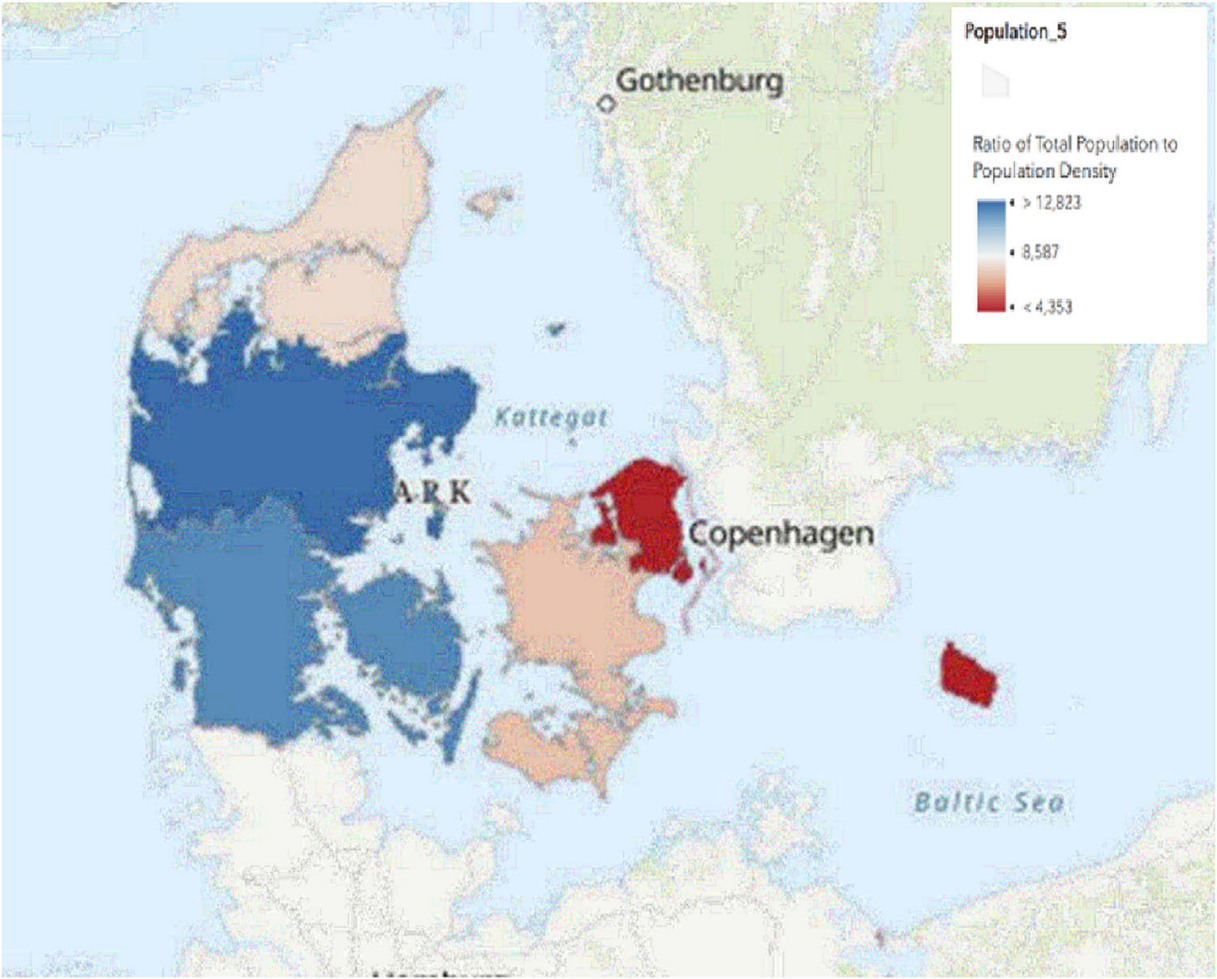
Ratio of population related to density.

Figure 1 details the landscape of future and new renewable energy infrastructure, which includes wind, solar, geothermal, sustainable heating, hydrogen, PtX, carbon capture, and e‐fuel projects. What is obvious is that not only in Zealand but also in Jutland, new infrastructure is planned or already built. This, on the one hand, is linked to the population – which is also seen in Figure 2 – but on the other hand, it resonates if it is considered that all major wind farms are built on the western side of the country or beyond (offshore).

Figure 2 illustrates the ratio of the population in the different regions in Denmark in relation to population density, as mentioned previously. The ratio of the total population to the population is presented.

**FIGURE 3:**
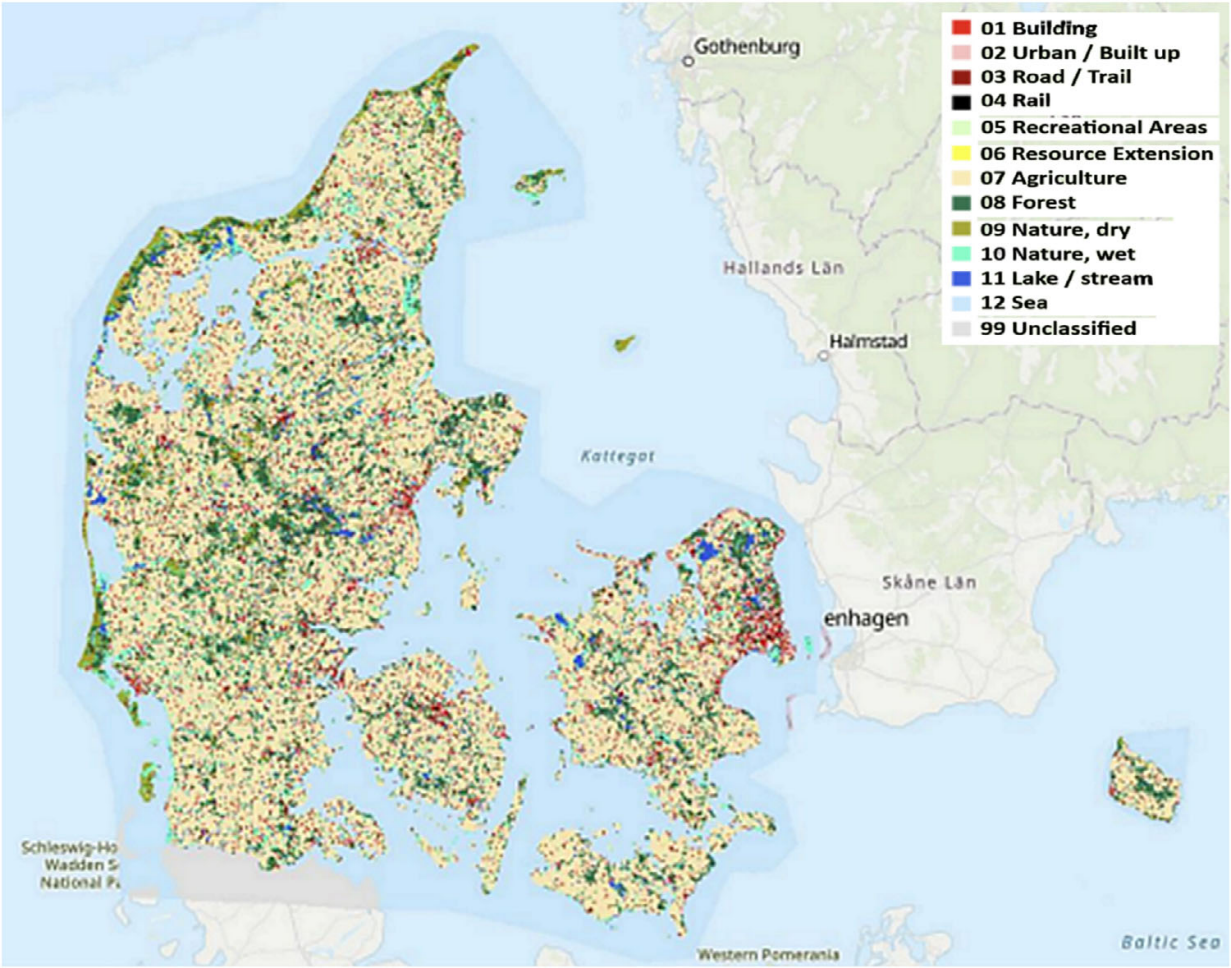
Land use base map [10].

Figure 3 presents the foundational land use base map, detailing existing urban, suburban, agricultural, and natural landscapes. Beyond the different types of land (or sea), infrastructure is taken into consideration here, such as roads and rails, and recreational areas. To systematically analyze spatial conflicts, specific analytical criteria were established within the GIS methodology followed. Proximity buffers of 500 meters (for noise/visual impacts) and 1,000 meters (for landscape/ecological disturbance) were applied around residential zones, schools, and protected natural areas. Overlap thresholds were defined to quantify land‐use competition; for example, projects overlapping more than 20% of high‐value agricultural land were flagged as high‐conflict zones. Conversely, sites within 2 kilometers of existing infrastructure were considered favorable, minimizing new needs (such as gas pipelines or transmission lines). These criteria provide a replicable framework for translating the visual layers into a quantifiable assessment.

**FIGURE 4:**
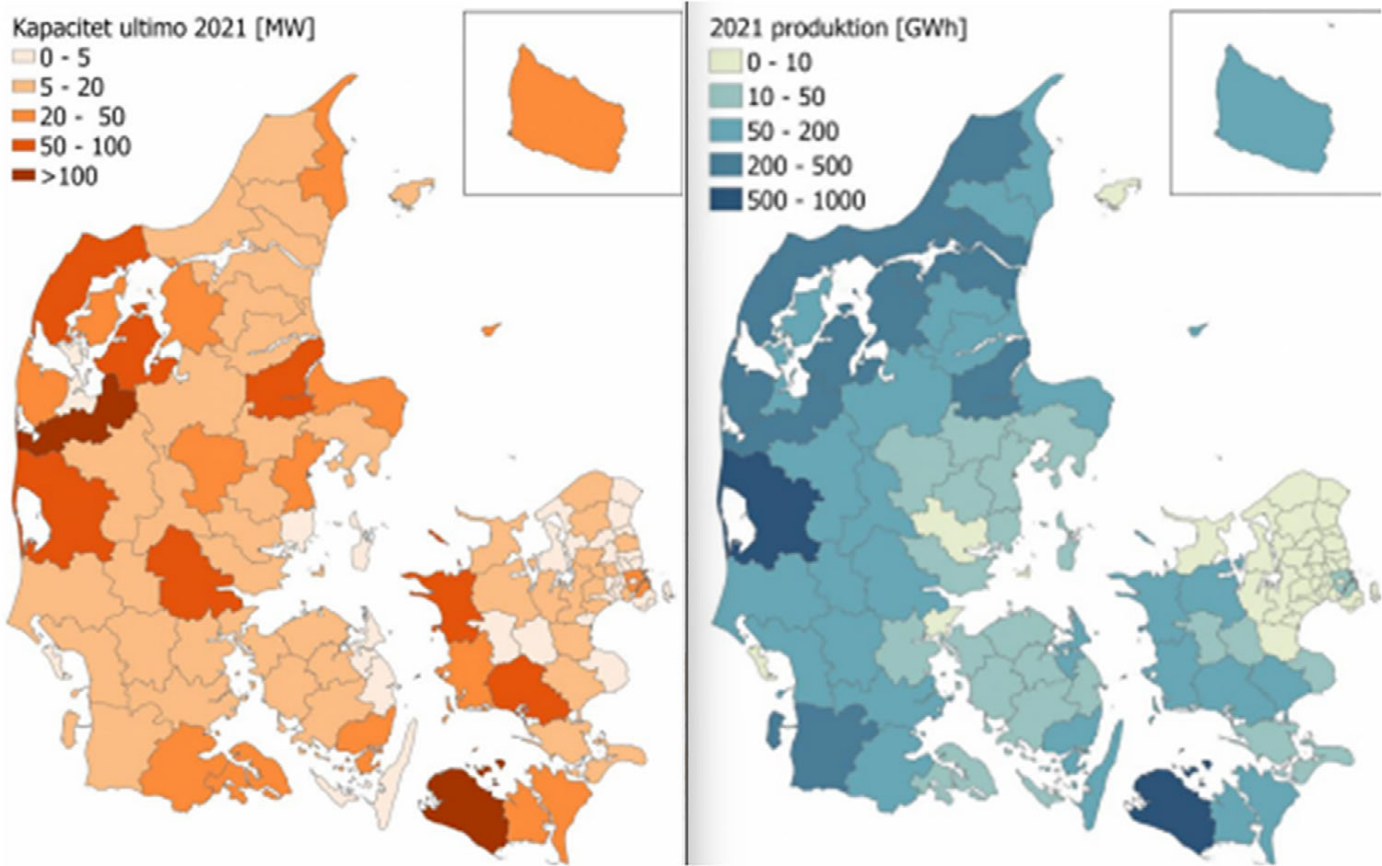
Solar MW capacity end of 2021 (left) & Map of production per municipality from onshore wind (right) [9].

This side‐by‐side analysis in Figure 4 highlights the geographic distribution and complementarity of two key renewable sources: solar capacity installed by municipality (left) and the tangible output of wind energy by municipality (right).

**FIGURE 5:**
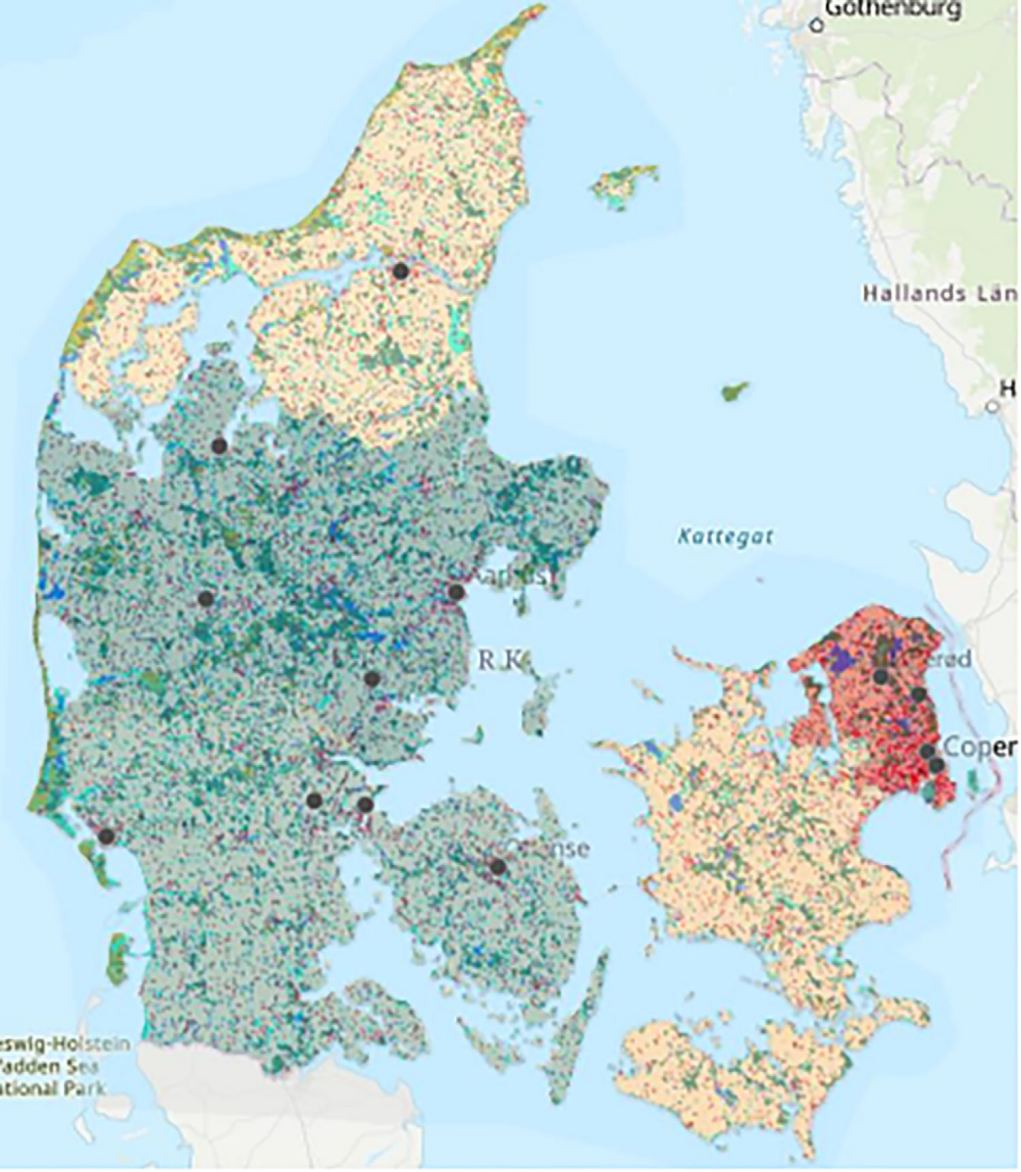
Combination of Figures 1, 2, and 3 showing land use, population density, and project sites.

Based on the GIS analysis, synthesizing all the layers, Figure 5 is the result. This composite map in Figure 5 provides a holistic spatial analysis by overlaying the project sites (Figure 1) with population density (Figure 2) and land use (Figure 3) base layers. Conflict zones shown are derived from the proximity buffers and overlap thresholds defined above.

## 8 Case Study Findings

### 8.1 Kassø PtX Facility: Southern Denmark

The Kassø PtX European Energy facility in Southern Denmark is one of the country's new green energy projects. This facility uses surplus power from offshore wind to produce green hydrogen through electrolysis, which is then converted into e‐methanol for ship fuel. A 50‐megawatt electrolyzer plant will be built, which will require substantial land use. The land use solution for this plant is highlighted for its integration into already existing industrial areas, reducing land conflicts. This land solution also avoids the need for new development on non‐industrial land, which also contributes to limited conflict regarding the plant's infrastructure [20]. More spatial focused strategies, such as the ones used in this case, could be helpful for solving other conflict issues in the future. The Kassø plant has also been involved with its local community by planning to use excess heat from the electrolyzer plant to power the local supply grid [21]. They have also engaged with local contractors, bringing in new jobs to the area [22].

### 8.2 Lille Torup: Jutland, Denmark

The Lille Torup project focuses on the development of a hydrogen transmission infrastructure in Jutland. This structure will connect a hydrogen storage facility in the area to other hydrogen production plants, including outside regions such as Germany. This hydrogen infrastructure is important to the development of Denmark's more hydrogen‐dependent energy grid. The construction of pipelines and storage facilities requires a great deal of land use, creating the possibility of land use conflicts. However, the developers for this project are attempting to strategically use existing pipeline areas and building locations to minimize disruption of the surrounding areas. This project is also working with the Danish Environmental Protection Agency to perform proper EIAs prior to any further permitting or construction, and an analysis of the possible socio‐economic effects [23]. Like the Kassø project, the Lille Torup project is attempting to bring economic benefits to the area through the creation of jobs. The steps taken by this project to minimize land conflicts and involve community leaders show a positive effort on the part of the developers to minimize any potential conflicts or injustices surrounding the project.

### 8.3 Fjord PtX: Western Denmark

The Fjord PtX project involves the development of a large e‐fuel production facility in Western Denmark. The facility will use energy from wind and solar farms to generate hydrogen, which is then converted into e‐fuels for different transportation sectors such as airline and shipping companies. Significant land use is needed for the development of electrolyzers, storage tanks, and conversion systems for fuel. Such a large‐scale project poses potential land conflicts with competing sectors in the surrounding area. The location of this plant is rural and has raised concerns from local communities about the possible effects on the surrounding natural land and the displacement of farmland. Developers are trying to mitigate these issues by choosing construction areas with little environmental value. Community engagement with this project has been somewhat resistant, as concerns are raised about environmental disruptions and displacement. This raises the question as to whether economic benefits from new job creation outweigh the negatives of possible environmental degradation and competition with farmers [24]. These case studies provide insight into the way Denmark is currently handling the challenges of PtX integration, with the conflicts that arise as a result. Each case provides a different viewpoint into the strategies used to handle these conflicts through strategized planning, community engagement, and social and environmental analysis [25]. As Denmark continues to grow its renewable energy transition using PtX technology, it is important to refine and develop new strategies for conflict mitigation. While detailed economic modeling was outside this study's scope, available project data reveal preliminary distributional patterns. The Kassø facility's surplus heat arrangement represents an estimated €2‐3 million annual value transfer to the local district heating grid [21]. The Lille Torup project publicly pledged approximately 50–75 construction and operational jobs for the surrounding region [23]. In contrast, the Fjord PtX project's benefit‐sharing mechanisms remain undefined, correlating with higher community resistance [24]. These figures, while not constituting full cost‐benefit modeling, illustrate the disparity in local economic integration across cases.

PtX projects in Denmark are critical to the country's strategy for decarbonization. The success of these projects not only depends on technical feasibility and appropriate funding, but also on public opinion, not only in Denmark, but also in the Nordic countries [26]. While national support for renewable energy is generally positive and in favor of expansion, local opposition from communities towards specific projects may create issues (Table 1). Community resistance has become a significant hindrance, with resistance halting 10–15% of green energy projects in Denmark [25, 27]. This resistance most often arises from concerns regarding environmental and social impacts. Locals often raise concerns about noise pollution, visual effects, and negative influences on property value (Table 2). It has been found that the construction of large‐scale PtX facilities can create a loss of agricultural land and alter community landscapes. The complexity of permitting and zoning may also create tension between local communities and developers [24].

Yet, some case studies, such as the Kassø PtX European Energy Facility, have been an example of proactive public engagement. In order to mitigate opposition to the project, European Energy decided to supply surplus heating to around 3,300 residents of the community. This solution focused on a dual beneficial integration approach by positively involving the local utility grid [24]. The Lille Torup project's use of existing pipelines is another example of positive integration, as this approach will aim to minimize the disturbance of the surrounding agriculture and environment. The project's focus on job creation is another example of creating benefits for both the developers and residents.

These approaches are crucial to the continual integration of PtX technology in Denmark. Through prioritization of community engagement, shared economic benefits, and open communication throughout the development process, PtX can find technological advancement and legitimacy (Table 3).

## 9 Discussion

This analysis offers two primary theoretical insights extending beyond existing PtX and energy justice literature. While PtX literature emphasizes technical and macroeconomic feasibility, this study empirically grounds PtX as a socio‐technical system, where the “X” also represents sites of potential social contestation over land use. Furthermore, this work applies energy justice frameworks – procedural, distributional, and recognition – to a novel industrial context. PtX introduces hybrid conflicts where rural industrial development compounds distributional injustices (i.e., agricultural land loss). A novel insight emerges from the stakeholder analysis: the mediating role of industrial legacy. The Lille Torup case demonstrates that repurposing existing infrastructure (“spatial continuity”) can significantly reduce opposition. This suggests that for industrial‐scale green technologies, the least conflictual path may be sensitive re‐use of brownfield energy landscapes rather than pristine greenfield sites. In this way, the findings from this study highlight the trade‐offs associated with the expansion of PtX technology within Denmark. The main problems focused on for this study were the effects of these technologies on Denmark's land use and social equity. The spatial analysis in Figures 1, 2, and 3 shows that PtX infrastructure is largely located in the rural areas west of the Copenhagen region. This is likely due to the availability of land and ideal conditions to produce renewable energy [28]. However, these areas often have lower population densities and less economic power, leading to higher risks of reinforcing existing inequalities within these regions. These areas have larger land availability, more ideal wind speeds, and closer proximity to transmission corridors, making them a more suitable choice for PtX facility development. Figure 2 shows the nation's population ratios, displaying the contrast between lower‐density rural areas and higher‐density urban areas. These rural areas are often host to large‐scale PtX development that unequally affects land use and the local landscapes of these regions. While urban areas benefit more from these projects as they tend to have higher energy demands and more economic growth, urban areas may gain more access to cleaner fuels and energy without facing many of the environmental and social costs. This observation is consistent with previous studies, such as in [6] and [8] that found rural communities face more intense land competition and environmental impacts from these projects. The case studies analyzed, such as Kassø, highlight these patterns even more. The Kassø facility chose a strategic location within an existing industrial brownfield, and the arrangement of supplying surplus heat to the surrounding area mitigated tensions between the host city and developers by providing community benefits. In contrast, the Fjord PtX project faced resistance due to concerns over changes in the landscape and the loss of farmland. The differences between the two cases show that if not planned appropriately, socio‐economic issues can be heightened.

The figures also showed that PtX projects often overlap with existing land uses, particularly farmland, as seen in Figures 3, 4, and 5. This competition risks disrupting local food production and heightening community pushbacks from residents and farmers [29]. This pattern was seen in the stakeholder analysis and previous literature from [7] and [9]. These papers argue that these dynamics must be mitigated through a more equitable distribution of the burdens associated with PtX projects. The Lille Torup project is an example of minimizing land use conflicts using existing pipeline infrastructure. The depiction in Figure 5 of wind energy production per municipality adds to the idea that energy transitions have historically been based more in rural areas of the country. The integration of the new Lille Torup project with existing infrastructure helps to reduce the impact of land use associated with these new projects. PtX technology significantly contributes to stabilizing Denmark's energy grid and supplying renewable heat. For widespread adoption, policies must ensure that these environmental and economic benefits, including local heat production, are distributed equitably across all communities. Figure 5 shows the overlapping elements of Figures 1, 2, and 3, further identifying that the more rural area of western Denmark is taking on the majority of PtX projects while most of its land use is either agricultural or forested.

The use of case studies in this paper demonstrates the dynamics seen within the spatial analysis. In Kassø, the strategic use of existing site infrastructure and the decision to share surplus heat with local communities have proven to be an effective strategy in mitigating local opposition and gaining support. The Lille Torup developers’ efforts to use previous pipeline corridors and work in tandem with the Danish Environmental Protection Agency on early environmental impact assessments also prove to be a solid strategy in reducing environmental degradation and community concern. In contrast to these two case studies, the Fjord PtX project has faced more resistance and local criticism. Concerns over loss of agricultural land, environmental disruption, and overall negative impacts on the community have highlighted the real need for participatory planning and open communication.

The stakeholder analysis further confirmed the observations made throughout this study. Local governments and residents rose as key players in the gatekeeping project development, as they have the most direct exposure to the impacts felt from them. Exclusion from decision‐making and communication between other stakeholders leads to resistance that can negatively impact the process of development and taint the project's public perception. The national government and project developers play a major role in determining the timeline of these projects, but can at times exclude the opinion of smaller stakeholders such as residents and local communities. The analysis of stakeholder interests and scope of influence suggests that early alignment and communication between these different groups are critical to strong integration of PtX and a positive perception in the public view.

The following policy recommendations from these findings include:

1. *Systemize early and continuous community engagement*: The success seen in the Kassø project and resistance in the Fjord PtX project show the need for early and continuing communication between local leaders and residents from larger players such as the developers and government agencies. Legally mandating structured communication between all parties will help to prevent conflicts and ensure that the projects are in line and respond to needs expressed by the local community [30].
2. *Use of existing infrastructure and land*: The spatial data and the Lille Torup case highlight the potential benefits of using brownfield sites and existing infrastructure to limit land competition. National planning frameworks should be implemented to prioritize the reuse of existing areas. Tax incentives should also be considered to encourage the reuse of existing land by developers [31].
3. *Strengthen local benefit frameworks*: Policies should be implemented that require PtX projects to deliver tangible benefits to host communities, such as surplus heat or energy, guaranteed jobs, etc. These policies may help to address the issue of energy injustice within rural areas [32].
4. *Enhancement of environmental (EIA) and social impact assessments (SIA)*: To reduce the environmental and social impacts of PtX projects, EIAs and SIAs should be used on all projects [33, 34]. These assessments will help to identify and address risks associated with each project and help to mitigate the problems before they become a major barrier to the project's success.
5. *Development of regional PtX knowledge hubs*: There should be the development of university‐industry knowledge transfer hubs coordinated with universities, local governments, and developers [35]. These hubs will provide transparent data and information on the impacts of current PtX projects on the affected communities. These communication policies will help to build trust between local communities and the parties overseeing PtX developments.

The spatial analysis, case studies, and stakeholder analysis reveal that PtX will be crucial for Denmark to reach its climate goals, but that integration must be managed delicately to prevent exacerbation of existing environmental and socio‐economic inequalities [36]. Community engagement, proper site selection, and policy that distributes both benefits and burdens of this transition equally will be key to a successful and long‐standing integration [37].

## 10 Conclusion

This study identifies the many challenges and opportunities involved in the integration and development of PtX technology as a part of Denmark's green energy transition. Spatial analysis showed that while rural areas often have ideal conditions for many PtX projects, they also face more of the challenges associated with them. Case studies of three PtX sites showed how dynamics between stakeholders manifest based on different approaches to site development. These findings varied between positive outcomes from community engagement and integration and negative resistance over varying public concerns. The stakeholder analysis confirmed that transparent communication and equal distribution of project benefits are critical components for successful implementation. The results concluded several policy recommendations that will be beneficial for project development going forward. These recommendations include early and inclusive engagement with host communities, the reuse of existing industrial sites, when possible, the use of EIAs and SIAs, and increased community education on PtX projects. By utilizing principles of inclusive governance and social equity in PtX policies, Denmark can make great strides in PtX integration and decarbonization in the future. Future research should apply formal economic modeling to this distributional question, quantifying the fiscal transfers, job distributions, and energy price effects that determine whether the green transition deepens or reduces existing regional inequalities.

## Conflicts of Interest

The authors declare no conflict of interest.

## Data Availability

The data that support the findings of this study are available on request from the corresponding author. The data are not publicly available due to privacy or ethical restrictions.
